# A 13 µW Analog Front-End with RRAM-Based Lowpass FIR Filter for EEG Signal Detection

**DOI:** 10.3390/s22166096

**Published:** 2022-08-15

**Authors:** Qirui Ren, Chengying Chen, Danian Dong, Xiaoxin Xu, Yong Chen, Feng Zhang

**Affiliations:** 1 The Key Laboratory of Microelectronics Device and Integrated Technology, Institute of Microelectronics of Chinese Academy of Sciences, Beijing 100029, China; 2 School of Integrated Circuits, University of Chinese Academy of Sciences, Beijing 100049, China; 3 School of Microelectronics, Xiamen University of Technology, Xiamen 361024, China; 4 The State Key Laboratory of Analog and Mixed-Signal VLSI and IME/ECE-FST, University of Macau, Taipa, Macao 999078, China

**Keywords:** analog front-end (AFE), EEG, RRAM-based lowpass FIR filter, ultra-low power, signal process, CMOS

## Abstract

This brief presents an analog front-end (AFE) for the detection of electroencephalogram (EEG) signals. The AFE is composed of four sections, chopper-stabilized amplifiers, ripple suppression circuit, RRAM-based lowpass FIR filter, and 8-bit SAR ADC. This is the first time that an RRAM-based lowpass FIR filter has been introduced in an EEG AFE, where the bio-plausible characteristics of RRAM are utilized to analyze signals in the analog domain with high efficiency. The preamp uses the symmetrical OTA structure, reducing power consumption while meeting gain requirements. The ripple suppression circuit greatly improves noise characteristics and offset voltage. The RRAM-based low-pass filter achieves a 40 Hz cutoff frequency, which is suitable for the analysis of EEG signals. The SAR ADC adopts a segmented capacitor structure, effectively reducing the capacitor switching power consumption. The chip prototype is designed in 40 nm CMOS technology. The overall power consumption is approximately 13 µW, achieving ultra-low-power operation.

## 1. Introduction

With the intersection and integration of many disciplines, such as information science, neurophysiology, medical electronic technology, and microelectronics, the research on bioelectrical signal recording for medical monitoring has formed a new research field. Especially with the continuous improvement of the CMOS integrated circuit, the high-performance, low-power bioelectric signal recording system has broad application prospects and rapid development. The bioelectric signal recording system can enable doctors to accurately monitor the electrophysiological activities of the human body such as brain waves and nerve signals in real-time and provide reliable monitoring data and indicators for doctors to accurately diagnose and treat patients. After many years of exploration and research, we have seen several well-established clinical bioelectrical signal recording systems such as electroencephalogram (EEG) signal recording, electrocardiogram (ECG) signal monitoring, urinary control, and functional nerve–muscle electrical stimulation, as well as in rehabilitation medicine, e.g., epilepsy and spinal injury. It plays an important role in improving people’s quality of life and improving people’s health [1,2,3,4,5,6,7]. 

The human brain is the most complex structure known so far. It is a comprehensive organ of complex functions. Elucidating the working principle of the brain is one of the most profound problems faced by modern science. EEG signal provides vast information for the diagnosis of neurological diseases. This paper presents the analog front-end (AFE) circuit for the implantable ultra-low-power EEG recording system. The AFE includes the front-end amplifiers, filters, and analog-to-digital converters (ADC). Prior works [8,9,10,11,12,13,14] have many research results on this aspect, but there are still many problems, such as the input impedance of the amplifier circuit being affected by the coupling capacitance, high requirements for bias circuits, poor linearity, and too high cost and power consumption. 

Considering the current issues, this paper designs an EEG-processing AFE circuit, consisting of a chopper modulation amplifier, a ripple suppression circuit, a RRAM-based lowpass filter, and an 8-bit SAR ADC. The core part of this work is the RRAM-based lowpass filter, FIR filter is an important tool for neural signal processing, and has been widely used in various biomedical applications, such as motor-imagery-based BMIs [15], epilepsy detection [16], and speech synthesis [17]. The design of FIR filters is one of the bottlenecks in conventional neural signal processing ASICs because of high power and delay [16,18]. In our system, the FIR filter can be implemented by a memristor array, which has the advantage of parallel analog computing so that the results of multiple FIR filters can be computed at one time, significantly reducing the computation power and delay. There have been proposals of designing FIR filters using a memristor crossbar structure; however, most of them remain on the simulation level [19,20], and the experimental demonstration so far has been limited to a single 6-tap FIR filter using only six memristors [21]. In this work, we experimentally implement a long-tap FIR filter on a memristor array, which is more useful in practical applications. As the filter coefficients are stored in the memristor array, the output currents under input voltages represent the filter results. The advantages of the RRAM-based lowpass filter are as follows: First, as a non-volatile memory, RRAM has been demonstrated to be highly efficient for in-memory computing. Second, RRAM could directly process analog signals, and parallel computing is also feasible in the form of cross-point arrays, significantly reducing the computation power and delay. Last, RRAM have been shown to be fast and highly scalable down to a few nanometers, which could enable high-throughput processing of large-volume neural signals. For the first time, an RRAM-based filter has been introduced in an EEG-processing AFE circuit, while meeting the functions, it also achieves ultra-low power consumption.

## 2. Proposed AFE Architecture

**Figure 1:**
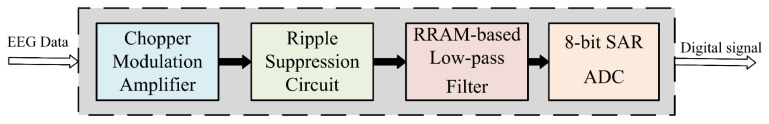
AFE diagram processing the EEG data input.

This work focuses on the implementation of the EEG AFE using the sub-blocks described in Figure 1. They are chopper-stabilized amplifiers, ripple suppression circuit, RRAM-based lowpass FIR filter, and 8-bit SAR ADC.

### 2.1. Chopper-Stabilized Amplifiers

EEG signal has the characteristics of small amplitude, low frequency, and easily corrupted with the environmental noise. To obtain EEG signals with a high signal-to-noise ratio, the AFE should have ultra-low noise characteristics. In the process of EEG signal acquisition, the noise source is mainly contributed by the flicker noise (1/f noise) of the device, which coincides with the operating frequency band of the EEG signal. To minimize the effects of the 1/f noise, chopper-stabilized amplifiers are inserted into the AFE circuit to eliminate 1/f- noise interference and suppress the DC offset voltages. 

**Figure 2:**
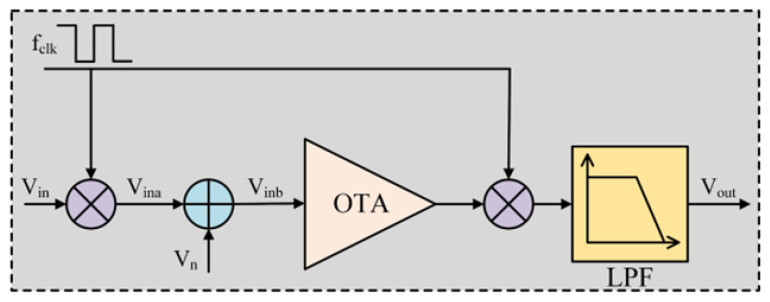
Flow chart of the chopper modulation signal.

Figure 2 shows the specific process of the chopper modulation technique. Herein, the chopper modulation scheme is merged into the fully differential amplifier to suppress low-frequency 1/f noise and offset voltage. The overall process of the chopper modulation is divided into modulation-signal, distortion-free, and amplification-demodulation. The signal conditioning first transfers the low-frequency EEG V_in_ to the chopping frequency spectrum (higher than the corner frequency of the device’s 1/f noise) through the first modulator, and then amplifies the transferred useful signal, low-frequency noise, and offset voltage at the same time. Then, the amplified signal travels through a second modulator, which modulates at the same frequency as the first. At this point, the low-frequency EEG V_in_ is demodulated back into the original frequency band, while the low-frequency noise and offset voltage are moved to the higher chopped spectrum. Finally, a succeeding lowpass filter is added to isolate the low-frequency EEG signals separately [22]. 

The input low-frequency EEG *V_in_* is first mixed with the first modulator operating at *f_clk_* (10 kHz in this work), *V_in_* is modulated to the odd harmonics of *f_clk_*. The output signal (*V_ina_*) is superimposed with the low-frequency noise and offset voltage (*V_n_*) of the amplifier input to obtain the signal *V_inb_*. Later, *V_inb_* is amplified by the amplifier and then mixed by the second modulator controlled by *f_clk_* to obtain the output signal (*V_out_*). 

**Figure 3:**
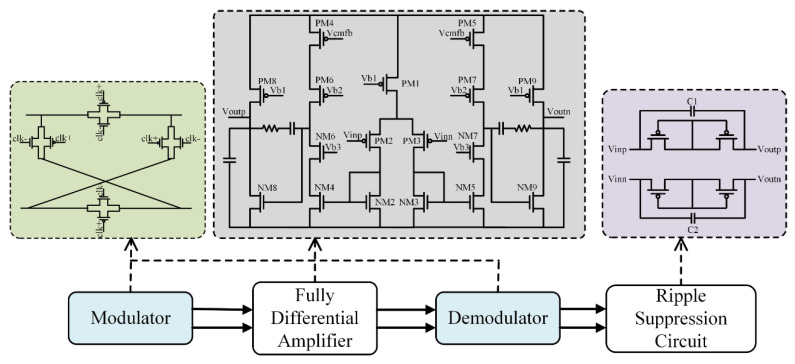
Ripple suppression circuit of the preamplifier.

The aforementioned modulator circuit can be realized by a switch circuit composed of the MOS transistors (Figure 3). It is controlled by two clock signals (clk+ and clk-) with the complementary phases to change the output of the MOS transistor to realize signal modulation or spectrum transfer. 

According to the design requirements of the EEG signal preamplifier, we designed a two-stage fully differential amplifier, meeting the needs of low noise and low power consumption, shown in Figure 3. The PM1 transistor is a current source transistor, which provides the quiescent current required for the normal operation of the op-amp. PM2 and PM3 are input pair transistors to amplify the input signal. The current mirror composed of NM2–NM5 acts as the load of the differential pair, and at the same time provides the current to the cascode stage to increase the resistance of the output terminal, leading to high gain. The first stage uses the symmetric OTA to increase the gain of the first stage through a cascode branch. The second stage uses a common-source stage loaded with a current source. Although the resistance of the common-mode feedback will reduce the output resistance of the second stage and affect its gain, because the gain of the first stage is larger, the gain of the second stage can be slightly sacrificed to meet other performances of the preamplifier.

### 2.2. Ripple Suppression Circuit

Once the chopping modulation technique is introduced into the circuit, the noise characteristic and the offset voltage has been improved. Yet, due to the offset voltage and 1/f noise of the preamplifier itself, it will superpose a ripple signal onto the output of the preamplifier after being modulated to the chopping frequency, which causes the output ripple signal to suffer from severe signal distortion. Its output ripple can be expressed as:

(1) $$V_{ripple,\;\;\;PP}=2V_{off}\left[\frac{\left(\frac{4}{\pi }\omega_{0}A_{0}\right)}{\omega_{CLK}}\cdot \frac{1}{{\left(1+\omega_{CLK}^{2}/\omega_{P}^{2}\right)}^{\frac{1}{2}}}\right]$$

where *V_off_* is the offset voltage at the output, *ω_0_* is the closed-loop bandwidth of the preamplifier, *A_0_* is the closed-loop gain of the preamplifier, $\omega_{P}^{2}$ is the bandwidth of the subsequent stage amplifier, $\omega_{P}^{2}$ = $\omega_{CLK}$ = 5*ω_o_*.

To suppress this distortion effect, a ripple suppression circuit is designed. The structure of this module is described in Figure 3. The main design ideas are as follows: The output ripple suppression circuit is connected to the parallel resistance–capacitance coupling circuit at the output end of the preamplifier. Considering the layout area, the resistance is generally realized by a MOS pseudo-resistor, which is formed by connecting two diode-connected MOS transistors in series. If the resistance is greater than the output impedance of the preamplifier, the RC coupling circuit will become an open circuit of the preamplifier at low frequencies. At the chopping frequency, if the impedance of the capacitor (at the chopping frequency) is less than the output impedance of the preamplifier, the RC coupling circuit acts as a short circuit. Therefore, at the chopping frequency, the output ripple signal of the preamplifier will not be able to pass through the parallel resistance–capacitance coupling circuit, achieving the purpose of suppressing the ripple signal [23,24]. The ripple amplitude at this time is:

(2) $$V_{ripple,\;\;\;PP}=\frac{2V_{off}}{G_{m1}R_{r}}\left[\frac{(\frac{4}{\pi })\omega_{0}A_{0}}{\omega_{CLK}}\cdot \frac{1}{{\left(1+\omega_{CLK}^{2}/\omega_{P}^{2}\right)}^{\frac{1}{2}}}\right]$$

where *G_m1_* is the output impedance of the preamplifier and *R_r_* is the resistance value of the pseudo resistor. For bio-signal amplifiers *G_m1_* ≈ 10 μA/V, *R_r_* ≈ 1 GΩ.

### 2.3. RRAM-based Lowpass FIR Filter

In the EEG signal conditioning circuit, the low-pass filter has the functions of suppressing out-of-band noise and anti-aliasing. Implementing programmable bandwidth control in the filter can expand the application range of the front-end circuit, making the front-end circuit adapt to different electrode types and different types of electrode implantation depths. In the process of EEG recording, we aim to reduce the power consumption of the system by reducing the sampling rate of the ADC when the EEG signal is normal. At this time, it is also required to lower the cutoff frequency of the low-pass filter accordingly to avoid aliasing of ADC samples. The array of RRAM with analog switching behaviors, where the device’s conductance can be continuously tuned, could carry out parallel analog computing. Thus, this allows us to run various signal processing algorithms while taking advantage of its high parallelism and efficiency in analog computing. In our system, the FIR filter is implemented by the RRAM array, which has the advantage of parallel analog computing, significantly reducing the computation power and delay. In this work, we experimentally implement a long-tap FIR filter on an RRAM array, which is more useful in practical applications. As the filter coefficients are stored in the RRAM array, the output currents under input voltages represent the filter results. Thus, an FIR filter with 40 Hz is designed and then implemented using the RRAM array to generate the waves in the corresponding frequency band.

A FIR filter can be mathematically expressed as [25]:

(3) $$y\left(n\right)=\overset{K}{\underset{k=0}{\sum }}x\left(n-k\right)h\left(k\right)$$

where *x* represents the input neural signal. *k* and *K* are the filter coefficient index and the filter order, respectively. *n* is the index of the time step. *h* represents the impulse response of the filter, whose pattern determines the property of the filter. *y* represents the filtered signal.

To implement the filter bank in the RRAM array, Equation (1) is re-written as follows [26]:

(4) $$y_{n}=x_{n}H$$

where *x_n_* and *y_n_* are the input and output signal row vectors in the nth time step, respectively. The matrix *H* represents the filter coefficients, and it is represented by the device conductance in an RRAM array for hardware implementation. Filter coefficients may have both positive and negative values; to solve this problem, we set two 1T1Rs for one weight, one for positive conductance, and the other for negative conductance. The conductance values of the two 1T1Rs are expressed as *Sp* and *Sn*, respectively. In this manner, the implementation of the filter in an RRAM array can be expressed as:

(5) $$I\left(n\right)=\overset{K}{\underset{k=0}{\sum }}\left\{V\left(n-k\right)\left(Sp\left(k\right)-Sn\left(k\right)\right)\right\}$$

where *V* is the input voltage vector and *(Sp(k) - Sn(k))* represents the mapped element of the k^th^ row of the filter coefficients matrix *H*. *I* is the output current vector of the filter.

**Figure 4:**
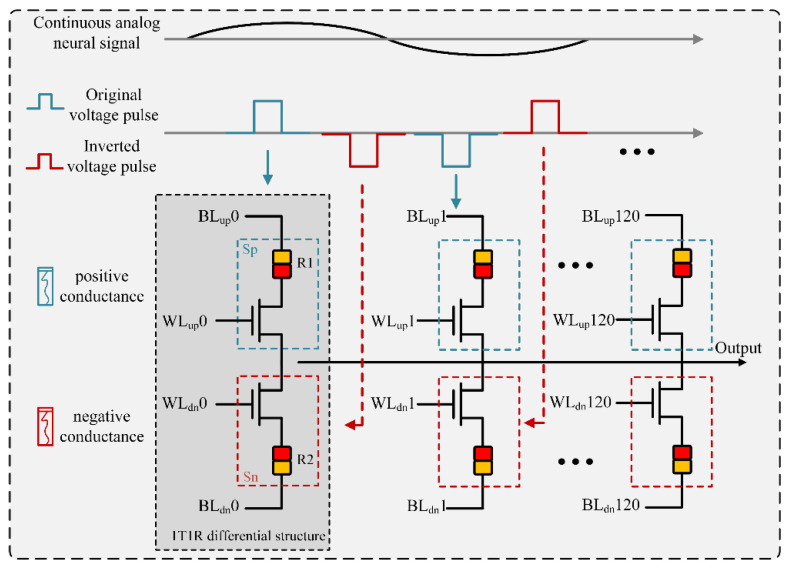
Implementation of the FIR filter for neural activities in an RRAM array.

Figure 4 shows how a neural signal is filtered in the RRAM array. For the 1T1R differential topology, the upper represents *S_p_* and the lower represents *S_n_*. When WL_up_ and WL_dn_ are high simultaneously, the two transistors are turned ON at the same time. BL_up_ and BL_dn_ are added with voltage values *V_sl_+V_i_* and *V_sl_-V_i_*, respectively, the current value flowing to SL is *V_i_/R_1_*- *V_i_/R_2_* by changing the positive and negative 1T1R conductance. Thus, the weight value of the weight unit can be changed. In RRAM arrays, 0.2 V(*V_i_*) is often used as the read voltage. Loading 0.2 V for a long time will not cause the resistance of RRAM to change. The coefficients of the designed filters are first mapped onto the RRAM array as the device conductance values. We set the filter order as 120. As a result, 242 RRAM devices are utilized to represent one filter with 121 coefficients. A clip of the analog voltage signal that contains the information of the brain state is then applied to the RRAM array. The sums of output currents are the filtered results from the filter at each time step. In this manner, the RRAM array filters out the input neural signals over the designed frequency band. The continuous analog neural signal is conditioned and sampled as the voltage pulses, which are fed to the input columns of the following RRAM array.

### 2.4. The 8-bit SAR ADC

**Figure 5:**
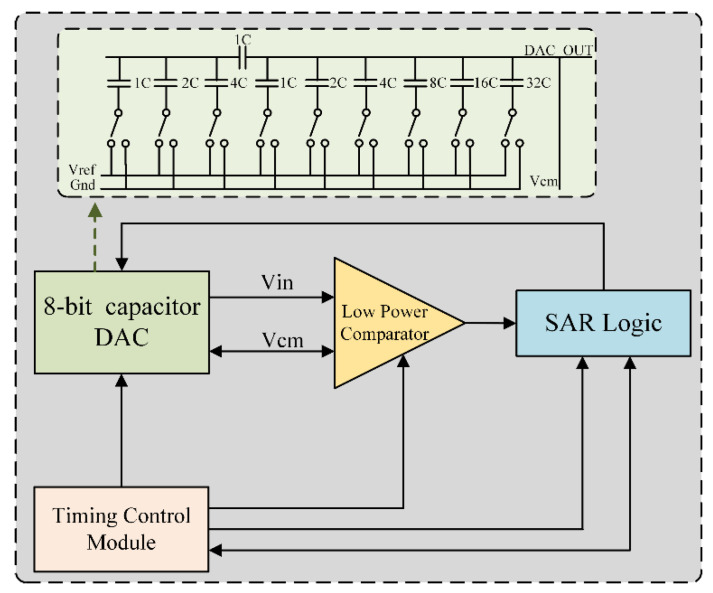
Block diagram of an 8-bit SAR ADC.

Figure 5 shows the block diagram of an 8-bit SAR ADC. It is mainly composed of an 8-bit segmented-capacitor digital-to-analog converter (DAC), a successive approximation register (SAR) logic circuit, and a comparator. In this structure, firstly, the sample and hold unit (i.e., in the capacitor-array SAR ADC, this unit can be merged into the capacitor-array module of the DAC) samples and holds the analog input signal (*V_in_*), as an input of the comparator unit. Then, the SAR unit starts the binary search algorithm until the digital code corresponding to the *V_in_* is obtained. Segmented capacitors (low 3 high 5) effectively reduce the total capacitance value of the capacitor array, which makes the DAC’s capacitor-switching power consumption drop sharply. Static errors are created in the SAR ADC due to the offset voltage in the comparator causing a DC drift in the comparator output. Therefore, the comparator adopts a three-stage preamplifier scheme with the input offset storage and output offset storage, which effectively reduces the influence of the DC offset voltage in the latch and amplifier.

## 3. Experimental Results

**Figure 6:**
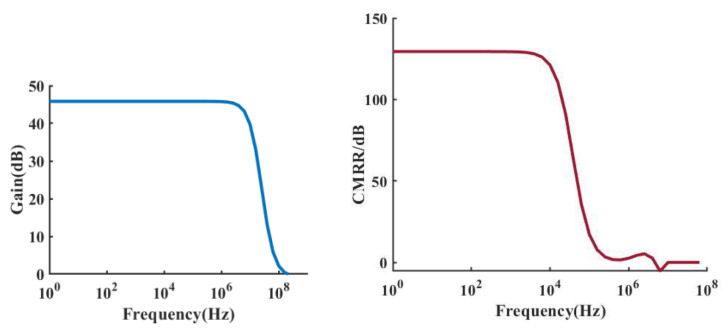
Gain and CMRR of the preamplifier.

**Figure 7:**
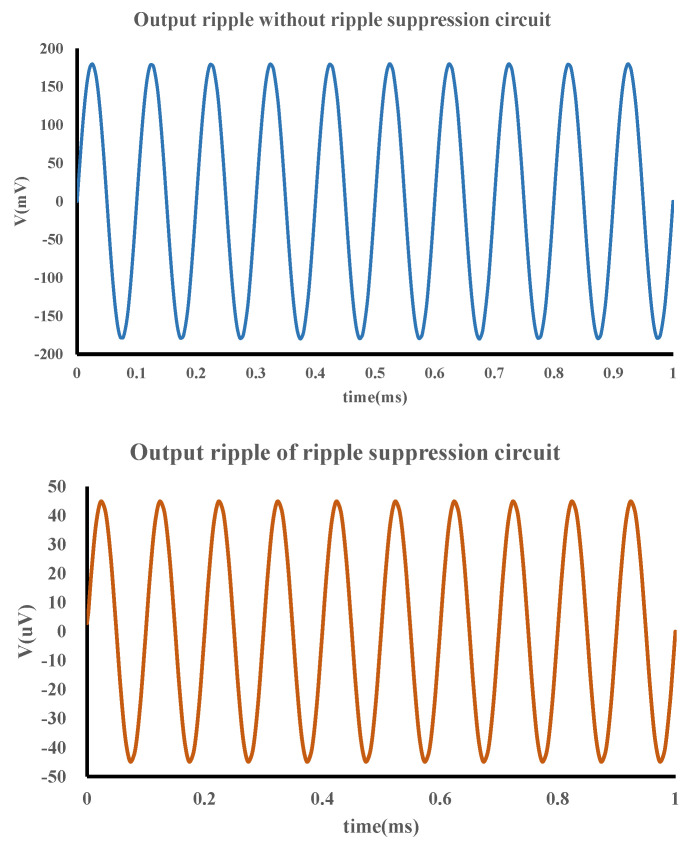
The output result of the ripple suppression circuit.

**Figure 8:**
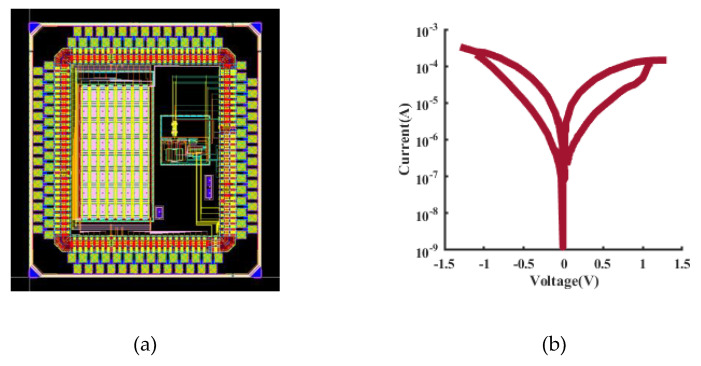
(**a**) Front-end circuit layout and (**b**) typical DC I–V curve for a single RRAM device.

**Figure 9:**
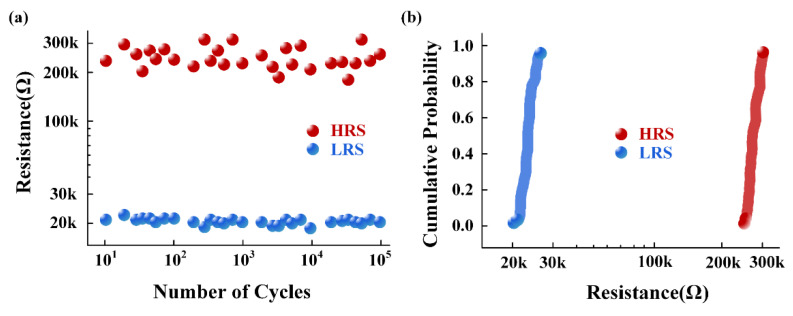
(**a**) The typical endurance performance of the 1T1R device and (**b**) HRS and LRS resistance stability measurement results.

The proposed AFE was fabricated in a 40 nm CMOS process. Figure 6 shows the simulation results of the gain and the CMRR of the preamplifier, which are 46 dB and 130 dB, respectively. The output offset voltage of the preamplifier was set to 1 mV and this offset voltage was used for transient simulations. As shown in Figure 7, the output ripple without the ripple suppression circuit is 360 mV, and the output ripple with the ripple suppression circuit is 90 μV, and the ripple is suppressed by 400 times. It can be seen that the ripple suppression circuit has a significant effect on reducing the output ripple of the preamplifier. Figure 8 depicts the layout of the front-end circuit and a typical DC I–V curve for a single RRAM device, showing excellent analog switching behaviors in both the SET and RESET processes. Figure 9a shows the endurance characteristic under pulse conditions. It is shown that the reversible resistive transition cycles are up to 10^5^ cycles without any observable degradation. As shown in Figure 9b, the cumulative probability of the HRS and LRS indicates that the device has little variation. The data are collected from 100 cycles of 30 randomly selected devices. 

**Figure 10:**
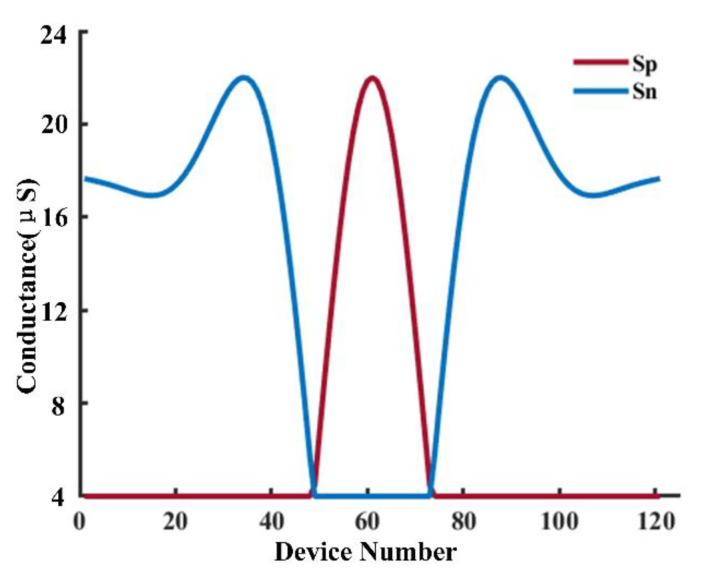
Conductance map for the lowpass FIR filter.

A 120th-order lowpass filter was designed, which requires a total of 242 RRAM devices, and a passband frequency ranges from 0 to 40 Hz. The conductance distribution is measured in Figure 10.

**Figure 11:**
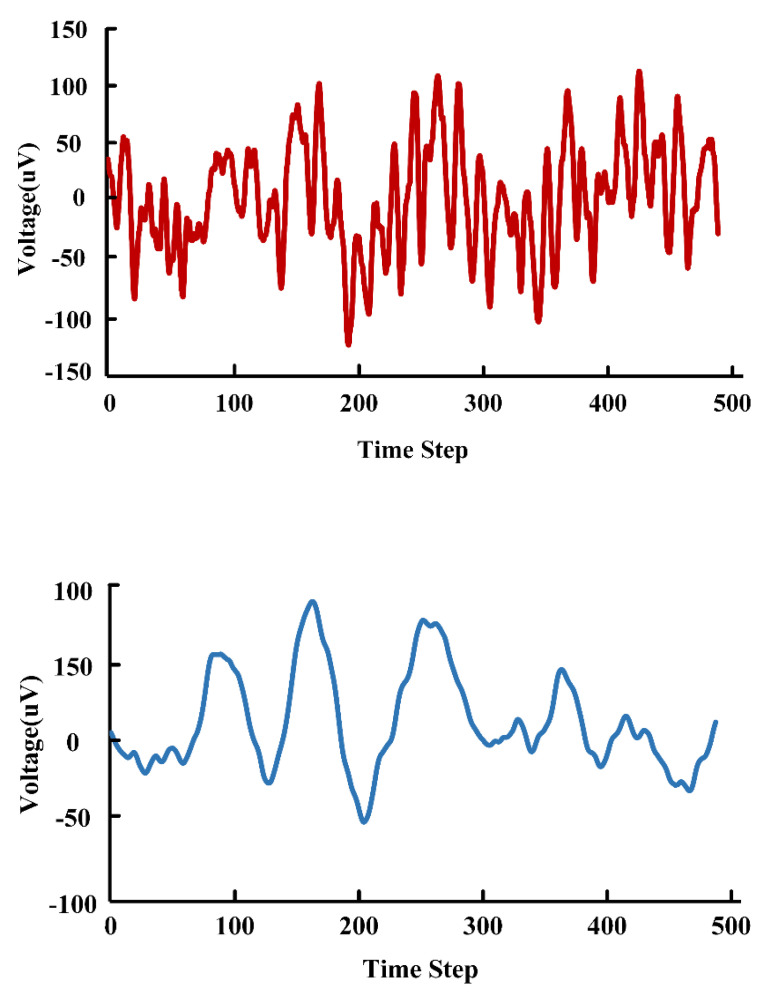
Simulated EEG raw data and filtered results.

For the system demonstration, we use the neural signals from the widely used Bonn Epilepsy Dataset. Figure 11 illustrates the original data and the result after filtering, respectively. It can be seen that the filter based on the RRAM achieves a good filtering effect.

A one-channel 0.1 *s* signal clip, which is sampled at 10 kHz, is the standard signal for power estimation. For a 1 *s* duration, there are 10 standard signal clips to be processed. The overall system power consumption is ~13 µW. The performance summary and a comparison with prior works are summarized in Table 1.

## 4. Conclusions

This brief presented an ultra-low-power AFE for the detection of EEG signal in the 40 nm CMOS process. The system was designed using four blocks for processing the acquired EEG signal, chopper-stabilized amplifiers, ripple suppression circuit, RRAM-based lowpass FIR filter, and 8-bit SAR ADC. The amplifier circuit provided a gain of 46 dB for the AFE. The cut-off frequency of the RRAM-based lowpass filter was 40 Hz. Taking advantage of RRAM for analyzing neural signals, the entire system achieved ultra-low power consumption of about 13 µW. 

## Figures and Tables

**Table 1:** Performance comparison with the published work.

	[14]	[13]	[12]	This Work
CMOS technology	25 nm	N/A	65 nm	40 nm
Gain	46 dB	48.5 dB	80 dB	46 dB
Low-pass filter	LC ladder network	RC network	N/A	RRAM-based FIR filter
Cut-off frequency	38 Hz	122 Hz	N/A	40 Hz
ADC	SAR ADC	NO	HAD-ADC	SAR ADC
Power consumption	32 μW	N/A	85 μW	13 μW

## Data Availability

The University of Bonn EEG database can be found at the following website: http://epileptologie-bonn.de/cms/upload/workgroup/lehnertz/ (accessed on 20 July 2022).

## References

[1] Lee T., Choi W., Kim J., Je M. Implantable Neural-Recording Modules for Monitoring Electrical Neural Activity in the Cen-tral and Peripheral Nervous Systems. Proceedings of the 2020 IEEE 63rd International Midwest Symposium on Circuits and Systems (MWSCAS).

[2] Shulyzki R., Abdelhalim K., Bagheri A., Salam M.T., Florez C.M., Velazquez J.L.P., Carlen P.L., Genov R. (2014). 320-Channel Active Probe for High-Resolution Neuromonitoring and Responsive Neurostimulation. IEEE Trans. Biomed. Circuits Syst..

[3] Das R., Moradi F., Heidari H. (2020). Biointegrated and Wirelessly Powered Implantable Brain Devices: A Review. IEEE Trans. Biomed. Circuits Syst..

[4] Lee J., Mok E., Huang J., Cui L., Lee A.-H., Leung V., Mercier P., Shellhammer S., Larson L., Asbeck P. An Implantable Wireless Network of Distributed Microscale Sensors for Neural Applications. Proceedings of the 2019 9th International IEEE/EMBS Conference on Neural Engineering (NER).

[5] Ng K.A., Yuan C., Rusly A., Do A.-T., Zhao B., Liu S.-C., Peh W.Y.X., Thow X.Y., Voges K., Lee S. (2019). A Wireless Multi-Channel Peripheral Nerve Signal Acquisition System-on-Chip. IEEE J. Solid-State Circuits.

[6] Lin Y.-P., Yeh C.-Y., Huang P.-Y., Wang Z.-Y., Cheng H.-H., Li Y.-T., Chuang C.-F., Huang P.-C., Tang K.-T., Ma H.-P. (2015). A Battery-Less, Implantable Neuro-Electronic Interface for Studying the Mechanisms of Deep Brain Stimula-tion in Rat Models. IEEE Trans. Biomed. Circuits Syst..

[7] Ts’O D.Y., Frostig R.D., Lieke E.E., Grinvald A. (1990). Functional Organization of Primate Visual Cortex Revealed by High Resolution Optical Imaging. Science.

[8] Harison R.R., Charles C. (2003). A low-power low-noise CMOS amplifier for neural recording applications. IEEE Trans. Solid-State Circuits.

[9] Qian C., Parramon J., Sánchez-Sinencio E. (2011). A Micropower Low-Noise Neural Recording Front-End Circuit for Epileptic Seizure Detection. IEEE J. Solid-State Circuits.

[10] Liu J., Zhang X., Hu X., Guo Y., Jialin L., Liu M., Li B., Chen H. (2015). A CMOS frontend chip for implantable neural recording with wide voltage supply range. J. Semicond..

[11] Ha U., Lee J., Kim M., Roh T., Choi S., Yoo H.-J. (2018). An EEG-NIRS Multimodal SoC for Accurate Anesthesia Depth Monitoring. IEEE J. Solid-State Circuits.

[12] Ren Y., He J., Liu J., Pan J., Wang X., Li C. An 8-Channel Wearable EEG Acquisition Front-End IC with Integrated Multi-Functions. Proceedings of the 2019 IEEE International Conference on Electron Devices and Solid-State Circuits (EDSSC).

[13] Diab M.S., Mahmoud S.A. 14:5nW; 30 dB Analog Front-End in 90-nm Technology for Biopotential Signal Detection. Proceedings of the 14:5nW; 30 dB Analog Front-End in 90-nm Technology for Biopotential Signal Detection.

[14] Morsalin S.M.S., Lai S.-C. Front-end circuit design for electroencephalography (EEG) signal. Proceedings of the 2020 Indo—Taiwan 2nd International Conference on Computing, Analytics and Networks (Indo-Taiwan ICAN).

[15] Higashi H., Tanaka T. (2012). Simultaneous Design of FIR Filter Banks and Spatial Patterns for EEG Signal Classification. IEEE Trans. Biomed. Eng..

[16] Abdelhalim K., Genov R. 915-MHz wireless 64-channel neural recording SoC with programmable mixed-signal FIR filters. Proceedings of the 2011 Proceedings of the ESSCIRC (ESSCIRC).

[17] Anumanchipalli G.K., Chartier J., Chang E.F. (2019). Speech synthesis from neural decoding of spoken sentences. Nature.

[18] Qaraqe M., Ismail M., Serpedin E. (2015). Band-sensitive seizure onset detection via CSP-enhanced EEG features. Epilepsy Behav..

[19] Mirebrahimi S.-N., Merrikh-Bayat F. (2014). Programmable discrete-time type I and type II FIR filter design on the memristor crossbar structure. Analog Integr. Circuits Signal Process..

[20] Nourazar M., Rashtchi V., Merrikh-Bayat F., Azarpeyvand A. (2018). Towards memristor-based approximate accelerator: Application to complex-valued FIR filter bank. Analog Integr. Circuits Signal Process..

[21] Alhammadi A.A., Nazzal T.B., Mahmoud S.A. (2016). A CMOS EEG detection system with a configurable analog front-end architecture. Analog Integr. Circuits Signal Process..

[22] Wei R.S., Zhu R. (2017). Low power chopper-stabilized amplifier. China Integr. Circuit.

[23] Mohammadpour A., Nabavi A. (2017). Design and analysis of a low-noise saw-less receiver front-end resistant to strong out-of-band blocker. Analog Integr. Circuits Signal Process..

[24] Hasan M.N., Lee K.S. (2015). A wide linear output range biopotential amplifie for physiological measurement front end. IEEE Trans. Instrum. Meas..

[25] Oppenheim A.V., Schafer R.W., Buck J.R. (1977). Discrete-Time Signal Processing.

[26] Liu Z., Tang J., Gao B., Yao P., Li X., Liu D., Zhou Y., Qian H., Hong B., Wu H. (2020). Neural signal analysis with memristor arrays towards high-efficiency brain–machine interfaces. Nat. Commun..

